# Benefits and limitations of sting challenge in hymenoptera venom allergy 

**DOI:** 10.5414/ALX02148E

**Published:** 2021-01-18

**Authors:** Katharina Aßmus, Markus Meissner, Roland Kaufmann, Eva Maria Valesky

**Affiliations:** Department of Dermatology, Venereology and Allergology, University Hospital, Goethe University, Frankfurt, Germany

**Keywords:** hymenoptera venom allergy, sting challenge, risk factors, therapy failure

## Abstract

The prevalence of systemic reactions to hymenoptera stings is up to 7.5%. Venom-specific immunotherapy (VIT) is an established treatment for insect venom allergy. In order to monitor the allergic status and thus the success of the therapy, controlled sting challenge under VIT continues to be the gold standard. This review deals not only with useful indications and therapeutic consequences but also with critical aspects that should be considered when performing sting challenge.

## Introduction 

Hymenopteran insect stings occur in up to 94.5% of the general population [1]. Clinical symptoms after an insect sting include physiological as well as allergic reactions, which may present as increased local reactions or systemic sting reactions (SSRs). The severity grade of SSR is highly variable and influenced by different factors. Mild reactions are usually generalized skin reactions such as flushing, urticaria, and angioedema. Dizziness, dyspnea, and nausea are considered to be moderately severe symptoms, whereas anaphylactic shock, unconsciousness, and respiratory/circulatory arrest are severe reactions. Epidemiological studies have shown that ~ 0.3 – 7.5% of adults and 3.4% of children are affected by SSR [2]. Venom-specific immunotherapy (VIT) is a highly effective treatment available to this patient population, with response rates of 77 – 84% in patients with bee venom allergy [3, 4] and 91 – 96% in patients with wasp venom allergy [3, 4]. The use of VIT is indicated when SSR exceeds skin symptoms or defined risk factors are present with previous mild SSR [5]. Patients receiving immunotherapy must have prior evidence of sensitization to the suspected insect [5]. Although the sensitivity of allergen-specific diagnostics for the detection of insect venom sensitization has significantly increased, no biomarker for therapy monitoring has been established [5]. So far, a controlled sting challenge is the only method that can reliably indicate presence or absence of venom tolerance, including clinical protection. Thus, a sting challenge under medical supervision remains the current gold standard, although it involves the apprehension to possibly expose the patient to an unnecessary risk. When considering the factors associated with an increased risk of SSR alongside a therapy failure, a sting challenge under medical supervision is considered to be more controllable than an accidental field sting. 

## Risk factors for systemic reactions under VIT 

Treatment with bee venom presents the most relevant risk factor, with an up to 6-fold increased risk of a systemic reaction [6, 7]. The previous assumption that mastocytosis and/or elevated serum tryptase might be similarly significant under VIT could not be confirmed with data from the current study. Only patients treated with wasp venom for elevated serum tryptase showed a slightly increased risk of systemic reactions (OR 1.56; CI 1.15 – 2.10) [6]. Patients with bee venom allergy were not affected by this phenomenon [8]. In addition, VIT for mastocytosis is considered to be a safe and effective treatment [9]. However, the extent to which concomitant medication with ACE inhibitors and β-blockers is considered to be an independent risk factor is currently under debate, with conflicting results [6, 10, 11]. 

Importantly, recent data have indicated that neither the severity of the index sting [6, 11, 12] nor the reaction threshold in the skin and the level of specific IgE can be considered risk factors for systemic reactions during immunotherapy [8, 12]. 

## Risk factors for recurrence of systemic reactions after termination of VIT 

Patients successfully treated with bee venom have a higher risk of developing a recurrence of their allergy than patients treated with wasp venom [13, 14]. Whether the severity of the index sting influences the likelihood of recurrence is controversial [15, 16, 17, 18]. However, the general consensus is that patients with an initially severe sting reaction are most at risk for the recurrence of SSRs [19]. Systemic reactions occurring during VIT are considered to be a relevant risk factor for a subsequent loss of protection (16.4 vs. 5.4%) [4]. The current data show that neither mastocytosis nor elevated serum tryptase is a relevant risk factor for an expected loss of tolerance [20]. However, it remains true that mastocytosis must be considered as a major risk factor in severe index reactions [5]. 

## Indication for sting challenge 

Current recommendations advocate a sting challenge under an ongoing VIT only. A controlled sting challenge is considered to be the gold standard to evaluate the clinical response of the patient [5], allowing a reliable conclusion about the success of allergen-specific immunotherapy [21, 22]. In a study of 129 patients, a reliable statement about the future tolerance of an experienced field sting could be made with the help of a sting challenge in 95% of the patients [21]. 

Patients who are at risk of future treatment failure (e.g., VIT with bee venom, systemic reactions during VIT, mastocytosis, index sting with severe reaction, concomitant medication with ACE inhibitors and β-blockers), despite ongoing VIT, benefit most from early knowledge of an insufficient protective effect of the VIT. By means of dose escalation, an effective protective effect can be achieved promptly in these cases [23]. 

Another benefit of a tolerated sting challenge is the improvement in disease-related quality of life, as the procedure can restore the lost sense of security experienced by severely impaired patients [24, 25]. 

## Contraindication 

Due to the increased risk of anaphylactic reactions as well as possible iatrogenic boosting of the allergy, sting challenges are not allowed in patients without VIT or after termination of VIT for routine reasons of evaluating clinical reaction status [26]. In addition, severe general and significant acute illness, uncontrolled bronchial asthma, poorly controlled hypertension, and pregnancy and lactation are absolute contraindications. If repeated systemic anaphylactic reactions occur at any time during maintenance therapy and until shortly before the planned sting challenge, a provocation should be avoided and, if necessary, an increase in the maintenance dose without the sting challenge can be performed [5, 26]. 

## Timing of sting challenge 

According to the EAACI recommendations, to detect treatment failure in a timely manner, a sting challenge should be performed as soon as possible after the maintenance dose is reached [5]. While it is unclear whether postponing the sting challenge to 6 – 18 months after reaching the maintenance dose, as recommended by some authors [26, 27, 28], reduces the likelihood of systemic reactions, this could be conceivable. Based on our own experience, we recommend a sting challenge 6 months after reaching and simultaneously tolerating maintenance therapy well. We consider performing a sting challenge at the end of VIT (after 3 – 5 years) to be less effective, as delayed detection of treatment failure is not only medically but also economically problematic. Special consideration should be given to the fact that sting challenges may cause a boost and, thus, lead to a relapse or an increase in the severity of the allergic reaction [26]. Studies have shown that in ~ 20% of the patients with a history of anaphylaxis and suspected venom allergy who received a diagnostic sting challenge, a systemic reaction did not occur until the second sting challenge; in some cases, these reactions were severe, i.e., required the use of catecholamines [29]. Therefore, to keep the possible booster effect low, if patients have almost finished their immunotherapy procedures, VIT should be continued for at least 6 months after the sting challenge [26]. After a tolerated sting challenge, VIT can be continued as soon as possible. It is important to keep in mind that a sting challenge does not replace therapeutic venom injection [26]. 

## Therapeutic consequences after a sting challenge 

**Figure 1:**
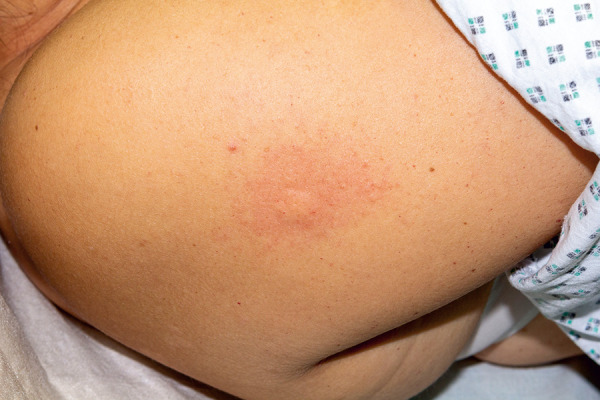
Sting challenge tolerated without systemic reaction: Only the physiologically expected wheal is visible. Symptoms distant from the sting were absent.

A tolerated sting challenge confirms therapeutic success and, thus, the effective treatment dose (Figure 1). However, the duration of VIT cannot be deduced from this alone. Research has shown that premature termination of VIT within the first 2 years of treatment results in a loss of tolerance with recurrence of systemic reactions in 22 – 27% of patients [30, 31]. To avoid a discussion with the patient about the premature termination of VIT, we recommend that patients be informed in advance about the rationale for sting challenge. In 2016, an EAACI task force discussed and reviewed whether patients should carry emergency medication with them after a tolerated sting challenge and the termination of immunotherapy is deemed unnecessary [32]. The continued prescription of epinephrine auto-injectors during and after VIT is recommended for children and adults with mast cell disorders, adults with index sting reactions beyond skin symptoms, and adults with risk factors for recurrent/severe SSRs. Based on the available data, carrying an emergency kit is recommended even after tolerated sting challenge. However, preventive use of emergency medications immediately after a field sting may be omitted if no symptoms are present aside from the sting itself [26]. 

If allergic symptoms besides the local sting reaction occur during a sting challenge, the maintenance dose should be increased in a venom-specific manner [23], and efficacy should be verified during the course by a repeated sting challenge [5, 23]. 

## Field sting or controlled sting challenge? 

**Figure 2:**
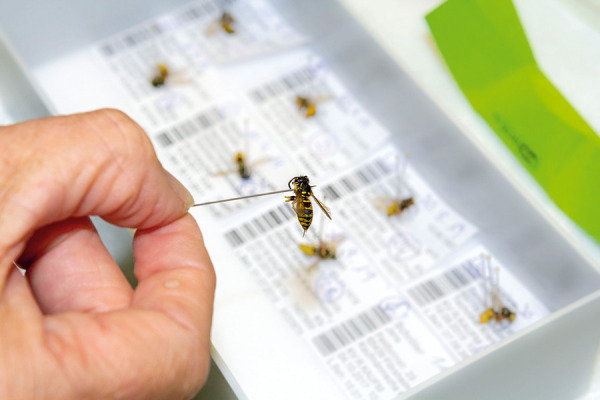
Entomological determination after sting challenge: Confusion between *Vespula sp. *and *Dolichovespula sp.* must be excluded.

The assumption that a tolerated field sting equates to a reliable occurrence of clinical protection is problematic for several reasons. First, accurate entomological identification (Figure 2) of the insect is usually not possible in the case of an accidental field sting, and patients often do not correctly identify the insect. Second, there is no way to ensure that the disease-causing insect actually stung the patient, as the approach of the insect is often misinterpreted as a sting. For example, it has been shown that a tolerated field sting after the end of immunotherapy is less meaningful than a tolerated sting challenge for assessing the sustained therapeutic effect [17]. If the patient has already reacted to a field sting with systemic allergy symptoms, this is considered as treatment failure and represents an indication to increase the VIT dose, even without a controlled sting challenge. The therapeutic success of the increased dosage needs to be further evaluated by a sting challenge. 

Sting challenges differ from other provocation tests because a stepwise application of the allergen in increasing doses is not possible [26]. The reluctance to perform sting challenges throughout Germany is certainly due to the erroneous assumption that it is an extremely risky exposure test. In a retrospective analysis of 1,609 sting challenges, Ruëff et al. [4] showed that with a lack of attention to risk factors, controlled sting challenges resulted in systemic reactions in 6.5% of the subjects. This low rate of anaphylactic reactions was confirmed by Kranert et al. [22], as systemic symptoms distant from the sting occurred in 1.5% of the provocations (15/968). While most reactions were not life-threatening, 2 bee venom allergy patients developed anaphylaxis during the sting challenge, which had to be treated with epinephrine [22]. In general, general allergy symptoms can be well controlled with immediate emergency care and are usually not life-threatening [26]. The situation is conceivably different if, in the case of an accidental sting event, patients are far from medical care and left to their own devices [26]. 

**Figure 3:**
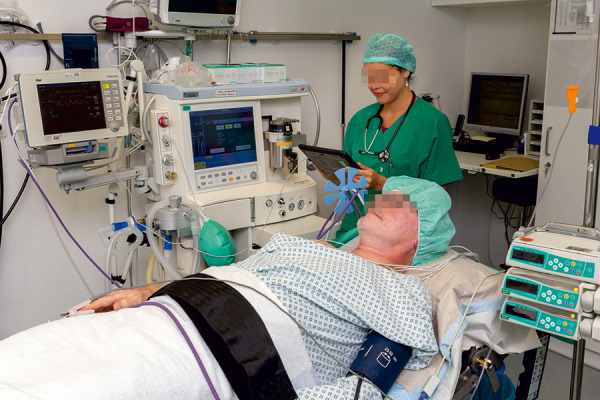
Initial monitoring in emergency preparedness during sting challenge.

Another reason for the reduced availability of controlled sting challenges throughout Germany is the requirement of technical and medical personnel. Sting challenges should be performed exclusively under emergency preparedness [26, 27]. The equipment and medication as well as the initial monitoring by medical personnel specially trained in emergency medicine are essential, thus complicating the implementation of this procedure (Figure 3). 

## Conclusion for practice 

Despite maximum safety precautions and standby of emergency management, there is a potential of severe anaphylaxis. With optimized patient preparation and elimination of transient risk factors, this risk can be minimized and is, thus, more controllable than an accidental field sting. If risk factors cannot be reduced, an increase of the maintenance dose up to 200 µg can be performed in high-risk patients, even without a prior sting challenge The aim of a sting challenge remains to verify the response and induction of tolerance during VIT. A tolerated sting challenge may also support the decision to resume a hazardous activity, especially via occupational exposure. The significant improvement in quality of life not only after initiation of VIT but especially after a tolerated sting challenge favors this procedure. Sting challenges without an ongoing VIT should be omitted because of the increased risk of severe anaphylaxis and the risk of boosting future allergic reactions. 

## Funding 

None. 

## Conflict of interest 

The authors declare no conflict of interest.
